# Extended Lymphadenectomy for Right-Sided Colon Cancer: A Systematic Review and Narrative Synthesis of Evidence, Technique, and Patient Selection

**DOI:** 10.7759/cureus.112512

**Published:** 2026-07-12

**Authors:** Dimosthenis Michelakis, Iraklis Perysinakis, Dimitrios Schizas, Maria Tolia, Paraskevi Karona, Eleni Manolessou, Eelco De Bree

**Affiliations:** 1 Department of Surgical Oncology, University General Hospital of Heraklion, Heraklion, GRC; 2 School of Medicine, University of Crete, Heraklion, GRC; 3 First Department of Surgery, Laikon Hospital, National and Kapodistrian University of Athens, Athens, GRC; 4 First Department of Radiology, Aretaieion University Hospital, National and Kapodistrian University of Athens, Athens, GRC

**Keywords:** central vascular ligation, complete mesocolic excision, d3 lymphadenectomy, lymph node metastasis, narrative synthesis, patient selection, right-sided colon cancer, systematic review

## Abstract

Right-sided colon cancer represents a substantial portion of all colorectal malignancies worldwide. Extended lymphadenectomy techniques, such as complete mesocolic excision (CME) with central vascular ligation (CVL) and D3 lymph node dissection, aim to improve oncological outcomes through enhanced nodal harvest and dissection along embryological planes. Despite two decades of investigation and six prospective studies, the survival benefit remains unproven in unselected populations, and patient selection criteria remain poorly defined.

This International Prospective Register of Systematic Reviews (PROSPERO)-registered systematic review (CRD420261334953) and structured narrative synthesis followed the Preferred Reporting Items for Systematic Reviews and Meta-Analyses (PRISMA) 2020 guidelines. Four databases were searched through February 2026. Two independent reviewers screened records and assessed study quality using the Cochrane Risk-of-Bias 2 (RoB 2), Risk Of Bias In Non-randomized Studies - of Interventions (ROBINS-I), and A MeaSurement Tool to Assess systematic Reviews (AMSTAR-2). Given the documented inability of seven published meta-analyses to resolve this question through statistical pooling, a structured narrative synthesis was performed. Fifty-five studies were included: four completed or interim-reported prospective trials, one ongoing randomized controlled trial (RCT), one international prospective observational cohort, seven systematic reviews and meta-analyses, and 42 comparative observational studies.

Extended lymphadenectomy consistently increased lymph node yield across all prospective trials (Grading of Recommendations Assessment, Development and Evaluation (GRADE): low-certainty evidence). The only completed randomized trial with published primary survival data did not meet its primary disease-free survival (DFS) endpoint, although a non-significant trend favoring extended dissection was observed. Additional prospective studies from Germany, Russia, and Italy have not demonstrated a universal survival benefit. Intraoperative vascular injury was modestly increased with extended dissection (GRADE: moderate-certainty evidence), without a significant increase in overall complications or mortality. Exploratory, hypothesis-generating subgroup analyses from the largest trial identified a potential benefit in patients with advanced nodal disease and lymphovascular invasion; however, this finding requires prospective validation.

Extended lymphadenectomy provides superior nodal harvest without a proven universal survival benefit. Current evidence supports an evidence-informed, stratified approach rather than a uniform policy: standard D2 dissection for cecal and early-stage ascending colon cancer and selective CME with CVL and D3 dissection for advanced-stage disease and for hepatic flexure or transverse colon tumors with high-risk features when performed in experienced centers. The proposed selection framework is interpretative and requires prospective validation. Mature survival data from ongoing trials are needed before definitive guideline recommendations can be made.

## Introduction and background

Right-sided colon cancer, comprising tumors of the cecum, ascending colon, hepatic flexure, and proximal transverse colon, accounts for approximately 40% of all colorectal malignancies worldwide [1], with evidence of a proximal tumor shift and rising incidence, particularly in younger patients over recent decades [2]. Surgical resection with adequate locoregional lymph node clearance remains the cornerstone of curative-intent treatment. The extent of that clearance, however, has generated sustained and unresolved international controversy, specifically whether dissection should extend beyond the standard intermediate (D2) level to the central nodes surrounding the superior mesenteric artery and vein (the anatomical territory defined as D3 or central lymph node dissection) [3,4].

The lymphatic drainage of the right colon follows its vascular supply through three sequentially more proximal nodal stations: paracolic nodes (D1, along the bowel wall and marginal artery), intermediate nodes (D2, along the ileocolic, right colic, and middle colic vessels), and central or main nodes (D3, around the origins of these vessels at the superior mesenteric vessels) [4,5]. The retrocolic fascial system and embryological planes that govern this dissection have been the subject of detailed anatomical re-examination in recent years [6-8]. This anatomical architecture provides the oncological rationale for extended dissection: occult metastases in central nodes, if left in situ, represent residual locoregional disease that may perpetuate recurrence regardless of systemic adjuvant therapy [9]. The critical clinical question is how frequently these central nodes harbor metastases and whether their removal is therapeutically beneficial or merely an anatomical exercise in a population where such involvement is rare [10,11].

The modern era of extended lymphadenectomy for colon cancer was inaugurated by Hohenberger et al. in 2009 [3], who formalized the concept of complete mesocolic excision (CME), a sharp dissection within the embryological plane between the mesocolic and parietal fascia, ensuring removal of an intact mesocolic envelope, combined with central vascular ligation (CVL) at the origin of the tumor-supplying vessels. West et al. subsequently demonstrated that CME with CVL produces an oncologically superior specimen compared with standard surgery [12]. Concurrently, and largely independently, Japanese surgeons had systematically developed D3 lymphadenectomy based on prospective anatomical mapping studies [4,10,13]. Despite sharing the same oncological objective, CME, CVL, and D3 dissection represent distinct but overlapping technical concepts that are frequently conflated in the literature, contributing substantially to the confusion surrounding their comparative efficacy [14,15].

**Figure 1:**
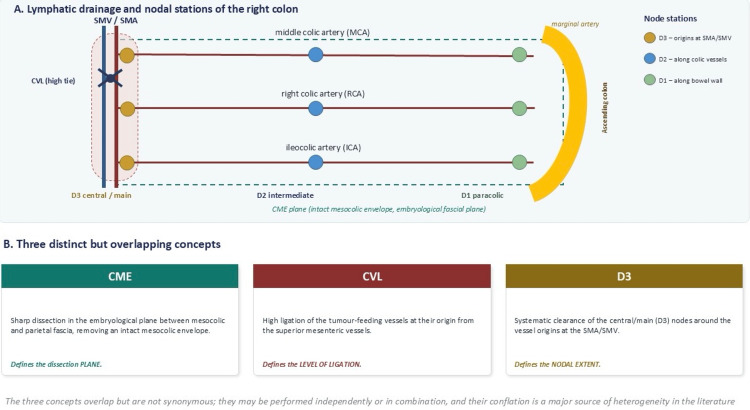
Schematic illustration of the D1-D3 nodal stations of the right colon and the distinction between complete mesocolic excision (CME), central vascular ligation (CVL), and D3 lymphadenectomy Panel A depicts the superior mesenteric vessels, the ileocolic, right colic, and middle colic vessels, and the D1 (paracolic), D2 (intermediate), and D3 (central/main) nodal stations, together with the CME dissection plane (dashed envelope) and the level of CVL (high tie). Panel B summarizes the three concepts. SMA: superior mesenteric artery; SMV: superior mesenteric vein Note: This image was created by the authors using Microsoft PowerPoint (Microsoft Corporation, Redmond, Washington, United States).

To orient the reader before the detailed treatment that follows, these three concepts are distinguished here in plain terms and illustrated schematically in Figure 1. CME defines the dissection plane (sharp dissection along the embryological fascial plane to remove an intact mesocolic envelope); CVL defines the level of vascular ligation (high tie of the tumor-feeding vessels at their origin from the superior mesenteric vessels); and D3 lymphadenectomy defines the nodal extent (systematic clearance of the central or main nodes around those vessel origins). The three are not synonymous and may be performed independently or in combination, a point developed further in the "Five Evidence-Derived Principles for the Rational Use of Extended Lymphadenectomy" section, Principle 3.

Despite widespread adoption in Asian surgical practice and growing enthusiasm in European and South American centers, prospective evidence supporting a survival benefit remains elusive. The RELARC trial (Radical Extent of Lymphadenectomy of LAparoscopic Right Colectomy), the only completed randomized controlled trial (RCT) with published primary survival data, found no statistically significant improvement in three-year disease-free survival (DFS) with CME + D3 compared with D2 dissection in 1,072 Chinese patients (HR 0.74, p=0.06) [16,17]. The RESECTAT/Benz study [18], a prospective non-randomized observational study involving 53 German centers, concluded that “no general benefit of CME could be established”; an exploratory finding in patients with stage III disease was characterized by the authors as being “of uncertain significance.” The COLD trial [19,20], conducted in Russia, has published only short-term safety data from its first 100 patients, while its primary endpoint of five-year overall survival (OS) remains unpublished. CoME-in (Complete Mesocolic Excision with Central Vascular Ligation in Comparison with Conventional Surgery for Right Colon Cancer) [21], the first Western multicenter phase III RCT, published an interim analysis demonstrating superior surgical quality metrics but no survival data, building on earlier feasibility trial results [22] (survival data expected January 2027). RICON (Comparison of D2 vs D3 Lymph Node Dissection for RIght COloN Cancer) [23] and the international T-REX prospective cohort [11,24] are ongoing or have recently been completed.

Multiple systematic reviews and meta-analyses have paradoxically deepened rather than resolved this clinical uncertainty. Seven published meta-analyses of overlapping observational evidence have reached contradictory conclusions [25-31], with pooled I² values ranging from 37% to 96%, reflecting irreducible clinical, methodological, and anatomical heterogeneity. Nevertheless, these analyses have provided important insights. They consistently demonstrated the increased nodal yield achieved with extended dissection and generated the hypothesis of a possible survival benefit, thereby helping to frame the clinical question. Our argument therefore concerns the limitations of further statistical pooling within this particular evidence base, which has tended to generate numerically precise but clinically misleading estimates, rather than the value of these reviews themselves.

This International Prospective Register of Systematic Reviews (PROSPERO)-registered systematic review (CRD420261334953) addresses four specific gaps in the existing literature: accurate characterization of all six prospective studies with validated risk-of-bias assessment [32-34]; Grading of Recommendations Assessment, Development and Evaluation (GRADE) certainty assessments for all primary outcomes [35]; integration of tumor location and feeding artery anatomy into a structured patient selection algorithm [11]; and formal justification of the narrative synthesis approach [36] through meta-epidemiological analysis of the prior meta-analytic literature.

This review aims to synthesize all available prospective evidence with accurate trial characterization and GRADE certainty assessment; identify patient, tumor, and institutional characteristics that define subpopulations most likely to benefit; explain why statistical pooling has failed to resolve this question; and propose an evidence-informed, three-tier patient selection framework for clinical consideration.

## Review

Methods

Protocol and Registration

This systematic review was conducted and reported in accordance with the Preferred Reporting Items for Systematic Reviews and Meta-Analyses (PRISMA) 2020 guidelines [37]. The protocol was prospectively registered in PROSPERO (registration number CRD420261334953; registered in March 2026). All eligibility criteria, search strategies, data extraction procedures, and synthesis methods were defined a priori. Methodological decisions were guided by the Cochrane Handbook for Systematic Reviews of Interventions (version 6.4) [36].

Eligibility Criteria

Population: Adults (≥18 years) with histologically confirmed right-sided colon cancer (cecum, ascending colon, hepatic flexure, or proximal transverse colon) undergoing curative-intent surgical resection were included.

Intervention: The intervention used was extended lymphadenectomy, including CME with CVL and/or D3 lymphadenectomy extending to central lymph nodes around the superior mesenteric vessels [3,4,12], performed via either an inferior or medial laparoscopic approach [38].

Comparator: Eligible comparator interventions included D2 lymphadenectomy (intermediate nodes along tumor-supplying arteries) or conventional right hemicolectomy without extended central dissection.

Outcomes: The primary outcomes were OS, DFS, and lymph node yield, while the secondary outcomes were perioperative complications (overall, vascular injury, anastomotic leak), operative time, local recurrence, and 30-day mortality.

Study designs: RCTs, prospective cohort studies, retrospective comparative studies with ≥50 patients per arm, and published meta-analyses were eligible. Single-arm case series (<50 patients), case reports, and editorials were excluded.

Information Sources and Search Strategy

We systematically searched PubMed/Medical Literature Analysis and Retrieval System Online (MEDLINE), Scopus, Web of Science, and the Cochrane Central Register of Controlled Trials (CENTRAL) from inception to February 28, 2026, without language restrictions. Search strategies combined Medical Subject Headings (MeSH) and free-text terms related to right-sided colon cancer, CME, D3 lymphadenectomy, CVL, and extended lymph node dissection. Reference lists of all included studies and relevant systematic reviews were also hand-searched. The complete database-specific search strategies are provided in Appendix 1 and in the PROSPERO protocol record (CRD420261334953).

Study Selection and Data Extraction

Two independent reviewers (D.M., I.P.) screened all titles and abstracts against predefined eligibility criteria using Covidence systematic review software (Veritas Health Innovation Ltd., Melbourne, Victoria, Australia). Full-text articles of potentially eligible records were independently assessed by both reviewers. Discrepancies were resolved through discussion or, when necessary, by arbitration from a third reviewer (D.S.). Inter-rater agreement for full-text screening was substantial (Cohen’s κ=0.84). Data extraction was performed independently and in duplicate using standardized, pilot-tested electronic forms. Extracted data included study design and setting, patient demographics and clinical stage, definitions of surgical interventions and quality metrics, primary and secondary outcomes with follow-up duration, and sources of funding. Inter-rater agreement for key extracted variables was also substantial (Cohen’s κ=[INSERT VALUE]). Any remaining discrepancies were resolved by consensus.

Risk-of-Bias Assessment

Risk-of-Bias (RoB) was assessed independently by two reviewers using validated tools appropriate for each study design: the Cochrane RoB 2 tool [32] for RCTs, Risk Of Bias In Non-randomized Studies - of Interventions (ROBINS-I) [33] for non-randomized and observational studies, and A MeaSurement Tool to Assess systematic Reviews (AMSTAR-2) [34] for included systematic reviews and meta-analyses. Full domain-level assessments for randomized and non-randomized studies are provided in Appendix 2; the AMSTAR-2 appraisal of systematic reviews and the risk-of-bias traffic-light summary are provided in Appendix 3.

Data Synthesis and Rationale for Narrative Approach

Rationale for narrative synthesis: A priori, we specified that quantitative meta-analysis would be performed only if at least three sufficiently homogeneous studies (I² < 50%) were available for a given outcome. The existence of seven published meta-analyses (2021-2025) analyzing substantially overlapping observational evidence, yet reaching contradictory conclusions and reporting I² values ranging from 37% to 96%, provided compelling evidence that additional statistical pooling would add confusion rather than clarity [25-31]. This observation constitutes a meta-epidemiological finding that supports the narrative synthesis approach [36]. We emphasize that these systematic reviews and meta-analyses were appraised using AMSTAR-2 and were included solely for contextual and meta-epidemiological evaluation to justify the narrative approach and characterise prior conclusions. They were not pooled with, or otherwise quantitatively combined with, the primary studies.

Heterogeneity domains precluding meta-analysis included intervention heterogeneity: definitions of CME varied substantially; some studies mandated specific mesocolic plane dissection and Benz specimen classification [18], whereas others required only CVL. The extent of D3 dissection (superior mesenteric artery versus superior mesenteric vein dissection) was also inconsistent across studies and geographic regions [14,15]. Outcome heterogeneity: thresholds for adequate lymph node yield (≥12, ≥15, ≥20, or ≥25 nodes), survival endpoints (three-year versus five-year; OS versus DFS), and follow-up duration (36-120 months) varied markedly. Population heterogeneity: stage distribution, definitions of tumor location, use of neoadjuvant therapy (0-43%), and patient selection criteria differed substantially. Methodological heterogeneity: study designs ranged from high-quality RCTs to retrospective single-institution comparisons with substantial confounding. 

A structured narrative synthesis was therefore performed, with results grouped according to study design hierarchy (RCTs > prospective cohorts > retrospective studies > meta-analyses); disease stage (I-II versus III); tumor location (cecum/ascending colon versus hepatic flexure/transverse colon); and geographic region.

GRADE Certainty Assessment

For all primary outcomes (OS, DFS, lymph node yield, and major complications), certainty of evidence was assessed using the GRADE approach [35]. Five domains were considered: risk of bias, inconsistency, indirectness, imprecision, and publication bias. Evidence was categorized as high, moderate, low, or very low certainty. GRADE assessments were performed independently by two reviewers. The full GRADE evidence profile is provided in Appendix 5.

Results

Study Selection

The systematic search identified 1,335 records across four databases (PubMed/MEDLINE: 450; Scopus: 380; Web of Science: 420; Cochrane CENTRAL: 85). After the removal of 285 duplicates, 1,050 unique records underwent title and abstract screening. Following this screening, 195 full-text articles were assessed for eligibility.

After full-text review, 140 articles were excluded for the following reasons: wrong intervention (n=45), wrong population (n=32), absence of an appropriate comparator group (n=28), insufficient extractable data (n=22), and duplicate publication from the same cohort (n=13). A complete list of excluded full-text studies and reasons for exclusion is provided in Appendix 4.

**Figure 2:**
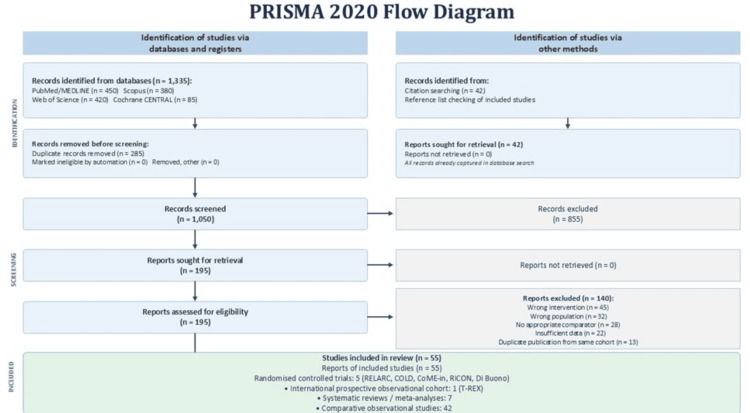
PRISMA flow diagram of the included studies PRISMA: Preferred Reporting Items for Systematic Reviews and Meta-Analyses; CENTRAL: Cochrane Central Register of Controlled Trials; MEDLINE: Medical Literature Analysis and Retrieval System Online; RELARC: Radical Extent of Lymphadenectomy of LAparoscopic Right Colectomy; CoME-in: Complete Mesocolic Excision with Central Vascular Ligation in Comparison with Conventional Surgery for Right Colon Cancer; RICON: Comparison of D2 vs D3 Lymph Node Dissection for RIght COloN Cancer

The final set of included studies comprised four completed or interim-reported RCTs [16-21], one ongoing RCT with a published protocol [23], one international prospective observational cohort (T-REX) [11,24], seven systematic reviews and meta-analyses [25-31], and 42 additional comparative observational studies. The PRISMA 2020 flow diagram is presented in Figure 2.

Evidence From Randomized Controlled Trials and Prospective Cohorts

Six prospective studies evaluated extended lymphadenectomy for right-sided colon cancer (Table 1). Of these, four are completed or interim-reported RCTs, and two are prospective observational cohorts with planned survival endpoints. Median follow-up is reported in Table 1, where available. For the COLD, CoME-in, and RICON studies, long-term endpoints remain immature or unpublished; therefore, survival outcomes should be interpreted within this temporal context.

**Table 1:** Characteristics of prospective studies evaluating extended lymphadenectomy for right-sided colon cancer CME: complete mesocolic excision; D2/D3: lymphadenectomy levels; DFS: disease-free survival; HR: hazard ratio; LN: lymph node; LVI: lymphovascular invasion; MCA: middle colic artery; NR: not reported; NS: not significant; OS: overall survival; SMA: superior mesenteric artery; RELARC: Radical Extent of Lymphadenectomy of LAparoscopic Right Colectomy; CoME-in: Complete Mesocolic Excision with Central Vascular Ligation in Comparison with Conventional Surgery for Right Colon Cancer; RICON: Comparison of D2 vs D3 Lymph Node Dissection for RIght COloN Cancer Median follow-up is given where reported by the source publication.

Trial	Country / Design	Sample (D2/D3)	Key findings	Median follow-up	Status
RELARC [16,17]	China (17 hospitals); Phase III RCT	1,072 (536/536)	3-yr DFS 86.1% vs 81.9% - HR 0.74 (0.54–1.02), p=0.06 NS. 3-yr OS 94.7% vs 92.6% - HR 0.70, p=0.17 NS. Vascular injury 3.1% vs 1.1% (p=0.045). pN2 subgroup HR 0.25 (p=0.001); LVI+ HR 0.34 (p=0.008).	~37 mo (verify)	Published
RESECTAT / Benz [18]	Germany (53 centers), prospective non-randomized	1,004	Primary conclusion: “no general benefit of CME.” Higher LN yield and Benz-0 specimens in the CME arm. Stage III exploratory (post-hoc) signal “of uncertain significance” per authors.	NR	Published
COLD [19,20]	Russia (4 hospitals); Phase III RCT	768 target; 100 published	Short-term only (n=100): 30-day morbidity 48% vs 47% NS. LN yield 27.8 vs 26.6 NS - limited surgical distinction between arms. 5-yr OS pending.	Immature (short-term)	Short-term
CoME-in [21]	Italy (9 referral centers); Phase III RCT (interim)	258 enrolled; 251 analyzed	Interim: LN yield 25 vs 20 (p=0.012). Mesenteric area 106 vs 98 cm² (p=0.04). Vascular injury 0.7% (n=1) vs 0%. Survival data expected January 2027.	Survival data expected in 2027	Interim
RICON [23]	Ukraine / Russia; multicenter RCT (protocol)	239 target	D2 vs D3 (NOT CME vs non-CME)-clearest surgical distinction. Stage II–III only. Recruitment completed September 2023.	NR (recruitment completed 2023)	Pending
T-REX [11,24]	7 countries / 36 centers; prospective observational	208 published (target 4,000)	Cecum: D3 metastasis 3.8%. Ascending colon: 1.1%. Transverse/MCA-fed: 6.7–7.5%. Routine SMA dissection is not required for the cecum/ascending colon.	Immature (interim)	Ongoing

Landscape of Published Meta-Analyses

We identified seven published systematic reviews and meta-analyses evaluating extended lymphadenectomy for right-sided colon cancer, published between 2021 and 2025 (Table 2) [25-31]. As described in the Methods, these reviews were appraised for methodological quality and used for contextual and meta-epidemiological evaluation rather than being pooled with the primary studies. Despite analyzing substantially overlapping evidence bases, they reached markedly different conclusions, illustrating the inherent difficulty of synthesizing heterogeneous surgical literature and supporting our narrative synthesis approach. At the same time, they were collectively consistent in demonstrating increased nodal yield and in generating the hypothesis of a survival benefit that the present review further examines.

**Table 2:** Key published meta-analyses and prospective observational studies on extended lymphadenectomy for right-sided colon cancer AMSTAR-2: A MeaSurement Tool to Assess systematic Reviews; CME: complete mesocolic excision; CVL: central vascular ligation; DFS: disease-free survival; GRADE: Grading of Recommendations Assessment, Development and Evaluation; JSCCR: Japanese Society for Cancer of the Colon and Rectum; LN: lymph node; MCA: middle colic artery; MD: mean difference; Mod: moderate; NS: not significant; OR: odds ratio; OS: overall survival; RoB: risk-of-bias

Study	Design / n	Key findings	I² / Quality	GRADE
De Simoni 2021 [25]	Meta-analysis; 8 studies, n=1,871	3-yr OS: OR 1.57 (p=0.003). 5-yr DFS: OR 1.99 (p=0.002). LN yield: MD +9.17 (p<0.001). Stage III OS: NS.	I²=37–96%; AMSTAR-2: Mod	Low
Hayashi 2024 [26]	Meta-analysis (stage-stratified)	CME survival benefit identified for Stage III only. No benefit Stage I–II. Supports a selective approach.	Stage-specific; AMSTAR-2: Mod	Moderate
Gupta 2024 [27]	Narrative review	CME+D3 selective, not routine. Cecum: CVL likely unnecessary. Transverse: stronger case. Learning curve 20–30+ cases.	N/A	Expert
Pompeu 2025 [28]	Meta-analysis	CME is associated with improved OS, DFS, and higher LN yield. No increased complications. Mostly retrospective evidence.	Mixed	Low
Tsukamoto 2023 (T-REX) [11]	Prospective observational; n=208	Cecum 3.8%, ascending 1.1%, transverse/MCA 6.7–7.5% main LN metastasis. Site-specific approach justified.	Low RoB	Moderate
Kataoka 2020 [10]	Retrospective JSCCR; n=4,034 Stage III	L3 metastasis: right 8.5% vs left 3.7%. Skipped metastases: 13.7% vs 9.0%. L3 is not an independent OS predictor in the right colon.	Moderate RoB	Low
Banipal 2024 [39]	Prospective subgroup; 42 D3+ of 623	D3-RDN R0 (n=29): 5-yr OS 72.9%, DFS 73.1%. Survival improves with experience. D3 metastasis is locoregional, not systemic.	Low–Mod RoB	Low

Quality assessment using AMSTAR-2 [34] demonstrated that, of the seven systematic reviews, two were rated as moderate quality, whereas five were rated as low or critically low quality. Common methodological limitations included the absence of a pre-registered protocol (5/7), inadequate assessment of publication bias (4/7), failure to account for study heterogeneity when interpreting pooled results (6/7), and inclusion of overlapping patient cohorts derived from the same databases (4/7).

Overall Survival and Disease-Free Survival

**Figure 3:**
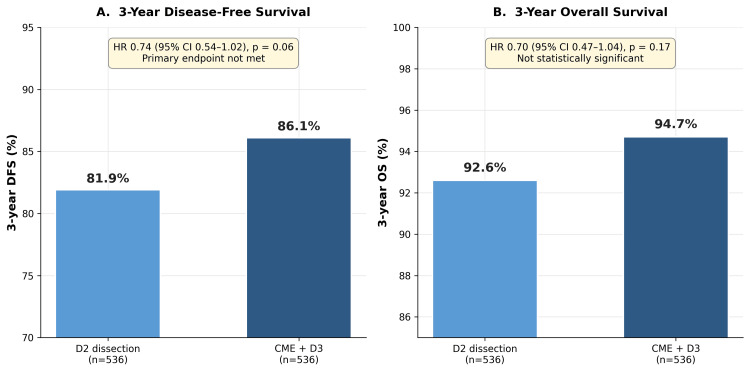
Primary survival outcomes from the RELARC trial (China, n=1,072) Panel A: Three-year disease-free survival, CME+D3 86.1% vs D2 81.9% (HR 0.74, 95% CI 0.54–1.02, p=0.06), primary endpoint not met. Panel B: Three-year overall survival, CME+D3 94.7% vs D2 92.6% (HR 0.70, 95% CI 0.47–1.04, p=0.17). Data visualized from Xu et al. [16] and Lu et al. [17]. RELARC: Radical Extent of Lymphadenectomy of LAparoscopic Right Colectomy; CME: complete mesocolic excision

Primary RCT evidence: Among the four prospective trials with published outcome data, only RELARC has reached its primary survival endpoint [16,17]. The results demonstrated a trend but did not achieve statistical superiority for CME+D3 over D2 dissection:

RELARC (JCO 2024) [16,17]: Three-year DFS 86.1% (CME+D3) vs 81.9% (D2); HR 0.74 (95% CI 0.54-1.02, p=0.06). Three-year OS 94.7% vs 92.6%; HR 0.70 (95% CI 0.47-1.04, p=0.17). Primary endpoint not met (Figure 3A). The confidence interval for DFS (0.54-1.02) is compatible with a clinically meaningful benefit, and the trial may have been underpowered to detect a modest true effect in an unselected population; a non-significant result does not, however, establish benefit.

RESECTAT/Benz (BJS 2023) [18]: Non-randomized prospective observational design. Primary conclusion: “No general benefit of CME could be established.” An unplanned, post-hoc exploratory subgroup analysis suggested a possible signal in Stage III patients, described by the authors themselves as “of uncertain significance” and requiring prospective validation.

COLD (BJS Open 2019; Br J Surg 2020) [19,20]: Only short-term data from the first 100 patients are available; long-term OS data (the primary endpoint) have not been published. Notably, lymph node yield was not significantly different between D3 (27.8 nodes) and D2 (26.6 nodes) groups, which may reflect the limited surgical distinction between arms in this trial.

CoME-in interim (ASO 2024) [21]: Survival data not yet available (expected January 2027). The pre-specified interim criterion (significantly higher LN yield in CME arm: 25 vs 20 nodes, p=0.012) was met, permitting continuation of enrolment.

GRADE LOW: Extended lymphadenectomy does not demonstrate a universal survival benefit across prospective trials. The only completed RCT (RELARC) did not meet its primary DFS endpoint. No pooled survival conclusion can be drawn from currently available prospective data.

Stage-stratified analysis: Significant heterogeneity exists in analyses stratified by disease stage, consistent with the hypothesis that benefit, if present, may be restricted to specific patient subgroups:

Stage I-II disease: No survival benefit was demonstrated in any prospective study [17,26]. The T-REX data [11] confirm that main lymph node (D3) metastasis rates are low in this setting: 3.8% for cecal cancers and 1.1% for ascending colon cancers, suggesting the therapeutic yield of extended dissection is limited in early-stage disease [40,41].

Stage III disease: An exploratory signal was observed in the RESECTAT/Benz non-randomized observational study [18] and in subgroup analyses of RELARC [17]. In RELARC, patients with pN2 disease (HR 0.25, p=0.001) and lymphovascular invasion (HR 0.34, p=0.008) demonstrated a potentially meaningful benefit from D3 dissection (Figure 3B); these were pre-specified subgroups analyzed in an exploratory, hypothesis-generating manner, whereas the RESECTAT/Benz Stage III signal was an unplanned post-hoc analysis. Both require prospective validation and should not be interpreted as definitive evidence of benefit.

GRADE VERY LOW: Stage III benefit from extended lymphadenectomy: signal observed in a non-randomized study and in exploratory RCT subgroups, but inconsistent across trials. Prospective validation is required before clinical recommendations can be made.

Observational evidence from meta-analyses: The seven identified meta-analyses [25-31] consistently reported improved three-year OS (pooled OR 1.57, p=0.003) and five-year DFS (OR 1.99, p=0.002) with CME in pooled observational data [25]. However, in the Stage III subgroup, where benefit is most expected, no significant OS difference was identified (OR 1.23, p=0.38). This paradox likely reflects selection bias, learning-curve effects, and confounding inherent to observational designs [42,43].

GRADE VERY LOW: Observational meta-analyses suggest a possible OS/DFS benefit with CME but are subject to substantial confounding and heterogeneity (I² 37-96%). Stage III subgroup analysis shows no significant benefit. Certainty is rated VERY LOW.

Lymph Node Harvest

**Figure 4:**
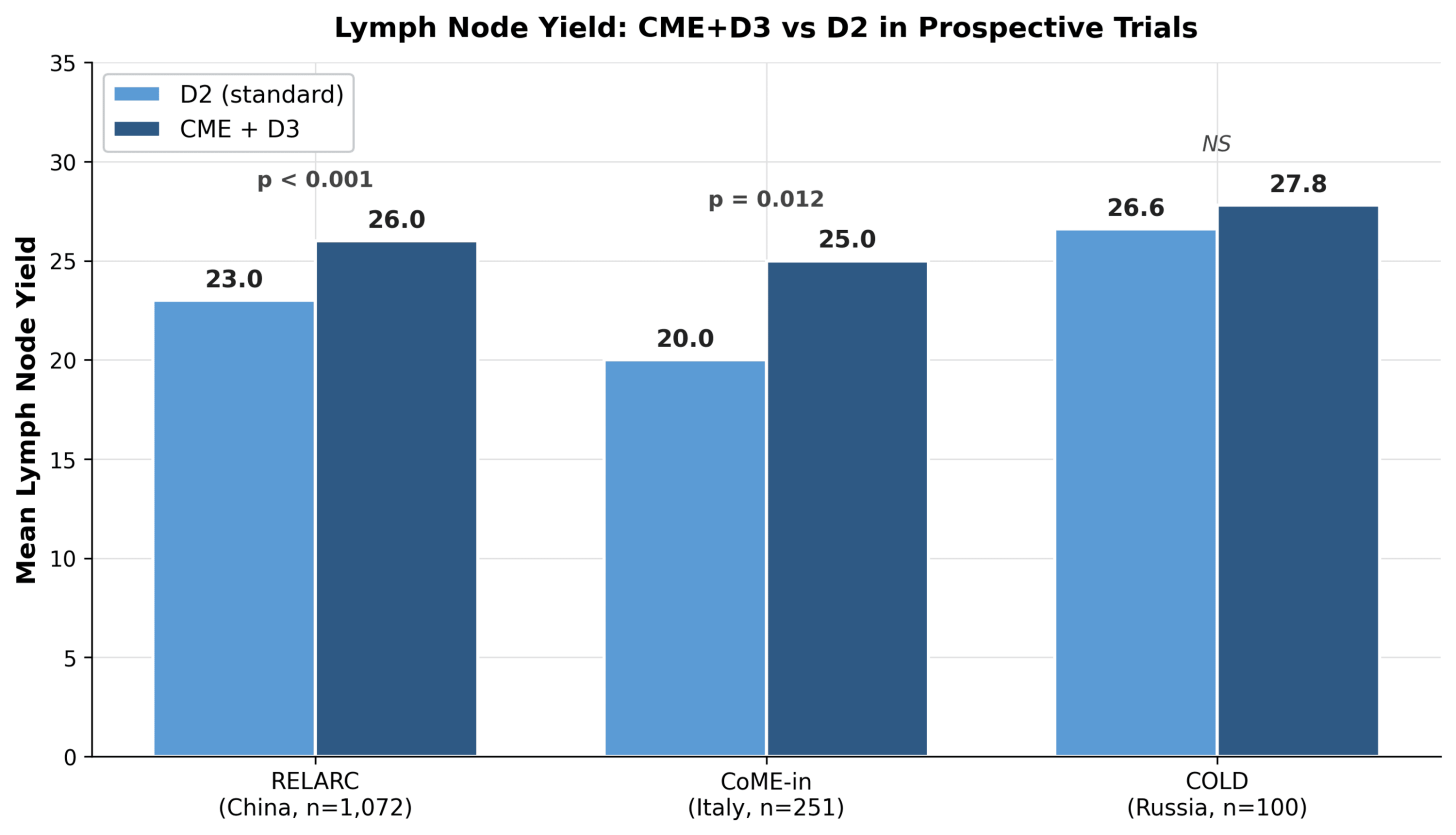
Lymph node yield in prospective trials of CME+D3 versus D2 dissection RELARC: 26.0 vs 23.0 nodes (p<0.001); CoME-in: 25 vs 20 nodes (p=0.012); COLD: 27.8 vs 26.6 nodes (NS). Data from Lu et al. [17], Karachun et al. [20], and Degiuli et al. [21]. RELARC: Radical Extent of Lymphadenectomy of LAparoscopic Right Colectomy; CoME-in: Complete Mesocolic Excision with Central Vascular Ligation in Comparison with Conventional Surgery for Right Colon Cancer; CME: complete mesocolic excision

Extended lymphadenectomy consistently and significantly increased lymph node yield across all prospective trials. RELARC [17]: 26.0 nodes (CME) vs 23.0 (D2), p < p<0.0001; mesenteric specimen area 116.4 vs 107.8 cm², p=0.001. CoME-in interim [21]: 25 nodes (CME) vs 20 (non-CME), p=0.012; ileocolic vessel length 13 vs 12 cm (p<0.001). RESECTAT/Benz [18]: higher yield in the CME arm with superior Benz specimen classification scores (76% Benz-0 in CME vs 22% in non-CME). COLD [19,20]: lymph node yield was similar between arms (27.8 D3 vs 26.6 D2, NS), suggesting the surgical distinction between arms may have been insufficient to generate a meaningful anatomical difference in this trial (Figure 4A).

The meta-analysis by De Simoni et al. [25] confirmed a pooled mean difference of +9.17 lymph nodes with CME (95% CI 4.67-13.68, p<0.001), though with very high heterogeneity (I²=96%). The clinical significance of increased nodal yield, beyond satisfying minimum adequacy thresholds, on survival outcomes remains unproven (Figure 4B) [5,26].

GRADE LOW: Extended lymphadenectomy significantly increases lymph node yield. Evidence is consistent across trials. Clinical impact on staging accuracy and survival benefit remains uncertain (downgraded for very high heterogeneity in pooled analyses and lack of survival correlation in RCTs).

Safety Profile and Perioperative Outcomes

**Figure 5:**
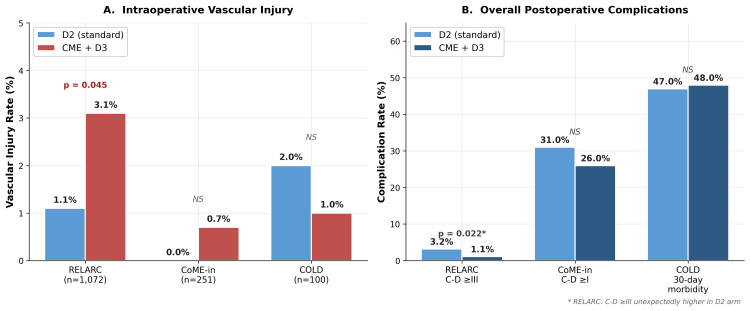
Perioperative safety profile (extended (CME+D3) versus standard (D2) lymphadenectomy) Panel A: intraoperative vascular injury rates, RELARC 3.1% vs 1.1% (p=0.045); CoME-in 0.7% vs 0% (NS); COLD 1.0% vs 2.0% (NS). Panel B: overall postoperative complication rates, RELARC Clavien-Dindo ≥III=1.1% vs 3.2% (p=0.022); CoME-in Clavien-Dindo ≥1=26% vs 31% (NS); COLD 30-day morbidity=48% vs 47% (NS). Data from Xu et al. [16], Lu et al. [17], Karachun et al. [20], and Degiuli et al. [21]. RELARC: Radical Extent of Lymphadenectomy of LAparoscopic Right Colectomy; CoME-in: Complete Mesocolic Excision with Central Vascular Ligation in Comparison with Conventional Surgery for Right Colon Cancer; CME: complete mesocolic excision

Vascular injury: The most clinically relevant safety signal associated with extended lymphadenectomy is an increased risk of intraoperative vascular injury. In RELARC [16], vascular injury occurred in 3.1% of CME patients (approximately 17 of 536) versus 1.1% of D2 patients (approximately six of 536) (p=0.045), predominantly involving the superior mesenteric vein and the gastrocolic trunk of Henle. In CoME-in [20], a single major vascular injury (0.7%, n=1, involving the SMA) occurred in the CME arm, with none in the non-CME arm. This finding is consistent across multiple observational series (Figure 5A) [43-46].

GRADE MODERATE: Extended lymphadenectomy increases intraoperative vascular injury risk, approximately 3% vs 1% (RR ~3.0). Most injuries are manageable intraoperatively. Risk is experience- and volume-dependent [42,47].

Overall complications and mortality: Despite the increased vascular injury risk, overall postoperative complication rates were not significantly different between extended and conventional dissection in any prospective trial. RELARC [16]: Clavien-Dindo ≥III 1.1% (approximately six of 536) vs 3.1% (approximately 17 of 536), p=0.022 (unexpectedly higher in the D2 arm); 30-day mortality 0% in both arms. CoME-in [21]: any Clavien-Dindo ≥1 complication 26% vs 31%, NS; 30-day mortality one per arm, NS; hospital stay shorter in the CME arm (p=0.039). COLD [19,20]: 30-day morbidity 48% vs 47%, NS; 30-day mortality 0% in both arms (Figure 5B).

Specific complications unique to extended dissection include chylous ascites (reported in 2.4% of D3 cases in Tsukamoto [11] and in early RELARC experience [48]) and lymphorrhea, attributable to division of lymphatic channels around the SMA. These complications are associated with the learning curve and appear to diminish with surgical experience [42,49].

GRADE MODERATE: No significant difference in overall postoperative complication rates, anastomotic leak [49], or 30-day mortality between extended and standard lymphadenectomy in prospective trials. Chylous ascites [47] and lymphorrhea are specific complications of extended SMA dissection that diminish with experience.

Lymph node metastasis patterns and implications for patient selection: The T-REX study [11,24] and the analysis by Kataoka et al. [10] provide critical anatomical data informing patient selection.

Tumor location as a determinant: Main lymph node (D3) metastasis rates vary significantly by tumor location and feeding artery. Cecal cancers (ileocolic artery (ICA)-fed) exhibit rates of ~3.8% at main nodes; ascending colon cancers (right colic artery (RCA)-fed) ~1.1%; but transverse colon/hepatic flexure cancers (middle colic artery (MCA)-fed) demonstrate rates of 6.7-7.5% around both SMV and SMA [11]. Anatomical variations of the SMA may further influence dissection planning [46].

Right vs left colon differences: Right-sided tumors more frequently demonstrate central (L3) node involvement (8.5% vs 3.7% in the left colon) and skipped metastases (13.7% vs 9.0%) [10]. Importantly, L3 involvement does not carry the same adverse prognostic significance in right colon cancer as in left colon cancer, suggesting a different biological relationship between nodal spread and systemic disease.

Central LN metastases are not necessarily systemic disease. Banipal et al. [39] demonstrated that patients with histologically confirmed D3 lymph node involvement who underwent R0 resection (D3-RDN group, n=29) achieved five-year OS of 72.9% and DFS of 73.1%, outcomes far superior to historical reports (five-year OS 35-48%), with survival improving over successive time periods, consistent with a surgical learning-curve effect [42]. These data derive from observational and subgroup analyses and remain susceptible to selection bias.

GRADE Summary of Evidence Certainty

Table 3 summarizes the GRADE certainty of evidence for all primary outcomes [35], incorporating risk of bias assessments (Cochrane RoB-2 [32], ROBINS-I [33], AMSTAR-2 [34]), inconsistency, indirectness, and imprecision across all included studies. Domain-level reasons for each downgrade are mentioned in Table 3.

**Table 3:** GRADE certainty of evidence summary (extended lymphadenectomy (CME/D3) for right-sided colon cancer) GRADE certainty: ⊕⊕⊕⊕ HIGH; ⊕⊕⊕○ MODERATE; ⊕⊕○○ LOW; ⊕○○○ VERY LOW. CME: complete mesocolic excision; DFS: disease-free survival; LVI: lymphovascular invasion; OS: overall survival; RCT: randomized controlled trial; RoB: risk of bias; RELARC: Radical Extent of Lymphadenectomy of LAparoscopic Right Colectomy Footnote: downgrading domains are stated within each row; full GRADE evidence profile in Appendix 5.

Outcome	Evidence base	GRADE	Interpretation
Lymph node yield	1 RCT + 7 meta-analyses	⊕⊕○○ LOW	Significantly increased with extended dissection. Clinical relevance for survival uncertain. Downgraded for inconsistency (I²=96%) and indirectness (yield is a surrogate not validated against survival).
3-year DFS (RELARC primary endpoint)	1 RCT (n=1,072)	⊕⊕○○ LOW	Trend favoring CME+D3 (HR 0.74) but p=0.06; primary endpoint not met. Downgraded for imprecision (CI crosses 1.0) and indirectness (single-country, unselected population).
5-year OS	Observational studies + meta-analyses	⊕○○○ VERY LOW	Reliable conclusions cannot be drawn from observational data. Downgraded for risk of bias (confounding/selection) and inconsistency (I² 37-96%).
Vascular injury	1 RCT + observational	⊕⊕⊕○MODERATE	Increased with extended dissection: ~3% vs ~1% (RELARC, p=0.045); experience-dependent. Downgraded once for imprecision (low event counts).
Overall complications	4 prospective trials	⊕⊕⊕○MODERATE	No significant difference between CME+D3 and D2 in any prospective trial. Downgraded once for imprecision.
Stage III subgroup benefit	1 RCT subgroup + 1 observational	⊕○○○ VERY LOW	Signal in pN2 (HR 0.25, p=0.001) and LVI+ (HR 0.34, p=0.008), RELARC subgroups only. Downgraded for indirectness (exploratory subgroups) and imprecision; requires prospective validation.

Discussion

This PROSPERO-registered systematic review synthesizes evidence from 55 studies, including four completed or interim-reported RCTs [16-21], one ongoing RCT [23], one international prospective cohort [11,24], and seven meta-analyses [25-31], to address the central clinical question of when and for whom extended lymphadenectomy provides oncological benefit in right-sided colon cancer. Our principal finding is that extended lymphadenectomy consistently improves lymph node yield but does not provide a universal survival benefit across prospectively studied populations. The evidence instead supports a stratified approach in which specific patient, tumor, and institutional characteristics, rather than a uniform surgical policy, should determine the extent of central nodal dissection.

The Central Paradox: Superior Nodal Harvest Without Universal Survival Benefit

The most consistent and reproducible finding across all four prospective trials is the significant increase in lymph node yield achieved with extended lymphadenectomy: from three additional nodes in CoME-in [21] to 13 in RELARC [17], compared with standard D2 dissection. This anatomical improvement is achieved without a commensurate increase in overall postoperative morbidity, anastomotic leak rates [48], or 30-day mortality when procedures are performed in experienced centers. Yet the RELARC trial, the only completed RCT with published primary survival data, demonstrates that this advantage does not translate into a statistically significant improvement in three-year DFS (86.1% vs 81.9%; HR 0.74, 95% CI 0.54-1.02, p=0.06) or OS (94.7% vs 92.6%; HR 0.70, p=0.17) in an unselected right colon cancer population [17]. The confidence interval is nonetheless compatible with a clinically meaningful effect, and the trial may have been underpowered for a modest true benefit; a non-significant result should not, however, be read as positive.

This apparent paradox, better nodal harvest, and no proven survival benefit, has several non-exclusive explanations grounded in the biology and anatomy of right-sided colon cancer. First, the therapeutic yield of central node dissection is limited in the majority of patients: main lymph node (D3) metastases occur in only 2.9-8.5% of unselected Stage II-III cases [10,11]. Second, right-sided colon cancers differ fundamentally from left-sided lesions at both the anatomical and molecular levels [2]: central node involvement does not carry the same independent adverse prognostic significance in right colon cancer [10], and the lymphatic drainage is anatomically more complex and variable than in the sigmoid or left colon [11,14]. Third, the COLD trial illustrated that when the surgical distinction between D2 and D3 arms is insufficient, reflected in virtually identical lymph node yields (27.8 vs 26.6 nodes) [19,20], no biological difference between treatment arms can be expected, regardless of the assigned intervention label.

Five Evidence-Derived Principles for the Rational Use of Extended Lymphadenectomy

Synthesis of the available prospective and observational evidence leads us to propose five operative principles that should govern the clinical application of extended lymphadenectomy for right-sided colon cancer.

Principle 1: Benefit is stratified by tumor and patient characteristics, not universal. The most important conclusion from the RELARC trial [17] is not the non-significant primary endpoint but the striking heterogeneity across pre-specified subgroups. Patients with pN2 disease derived a potentially meaningful DFS benefit (HR 0.25, 95% CI 0.11-0.57, p=0.001), as did those with lymphovascular invasion (HR 0.34, p=0.008). These pre-specified subgroups were analyzed in an exploratory, hypothesis-generating manner and require prospective validation in enriched populations before they can guide practice. The T-REX study [11] demonstrates that tumor location determines D3 node metastasis rates across a seven-fold range, from 1.1% in ascending colon cancers to 7.5% in transverse colon/MCA-fed tumors, directly supporting differential dissection strategies by location. The meta-analysis by Hayashi et al. [26] provides further corroboration: a significant survival impact of CME was identified only for Stage III disease. Predictive nomograms incorporating clinicopathological features may aid the identification of patients at higher risk for D3 metastasis.

GRADE LOW: Stratified benefit of D3 in pN2/LVI+ subgroups and MCA-fed tumors. Consistent biological rationale, but derived from exploratory subgroup analyses; requires prospective validation in enriched populations.

Principle 2: Surgical quality must be standardized before extent can be evaluated. A fundamental insight from the CoME-in interim analysis [21] and the RESECTAT/Benz study [18] is that the quality of mesocolic excision, measured by Benz specimen classification, mesenteric area, and vessel stump lengths, varies substantially even within experienced centers and that quality metrics are more reproducible and clinically informative than lymph node yield alone. In CoME-in, 76% of CME specimens achieved a Benz score of 0 versus only 22% of non-CME specimens [21], a finding of greater surgical significance than the difference in raw node counts. The Benz classification [18], photographic documentation of the specimen [50,51], and standardized vessel stump measurement [43] represent the minimum quality indicators that should accompany lymph node yield as a composite endpoint in future trials, and the Radical Right Colectomy-Surgical Technique Approved Report (RRoC-STAR) Delphi consensus [15] and standardized reporting frameworks [14,52] provide the infrastructure for valid inter-institutional comparisons. Importantly, these core metrics are low-cost and feasible outside high-volume centers: standardized photography, Benz grading, and stump/mesenteric-area measurement require no advanced infrastructure, and central pathology review with brief structured training improves inter-observer reproducibility, although grading reliability is itself experience-dependent and benefits from centralized review.

GRADE MODERATE: The quality of mesocolic excision (Benz classification) is independently reproducible, assessable, and feasible to implement in lower-volume centers. Recommended as standard reporting in all future trials.

Principle 3: CME, CVL, and D3 are distinct concepts that must not be used interchangeably. A pervasive source of confusion in the literature and a major explanatory factor for the contradictory conclusions of seven published meta-analyses is the interchangeable use of three technically and conceptually distinct terms (CME, CVL, and D3 lymphadenectomy), as illustrated in Figure 1 [14,15,53]. CME, as defined by Hohenberger [3,53] and refined in subsequent procedural descriptions [54,55], refers to sharp dissection within the embryological plane between mesocolic and parietal fascia, with removal of an intact mesocolic envelope. CVL refers specifically to high vascular ligation at vessel origins. D3 dissection, as defined by the Japanese Society for Cancer of the Colon and Rectum (JSCCR) guidelines [56], refers to the systematic removal of central lymph nodes around the superior mesenteric vessels, without necessarily mandating embryological plane dissection.

These three concepts can be performed independently or in combination, and their technical definitions vary across regions. Japanese D3 dissection historically focused on SMV-level dissection [4], while European CME with CVL extends the dissection further [12]. The COLD trial included all colon cancer sites and applied CME to all patients in both arms, meaning the only variable was D2 versus D3 node dissection extent, a fundamentally different comparison from RELARC, which compared CME+D3 against CME+D2 in laparoscopic right hemicolectomy [17,19,20]. Without recognizing these distinctions, pooling of these trials, as has occurred in multiple meta-analyses [25-31], is methodologically unjustifiable and clinically misleading. Sica et al. [14,15] and recent technical anatomical analyses [56] have provided the definitional framework necessary to resolve this confusion.

GRADE LOW: Definitional heterogeneity identified as the primary driver of contradictory meta-analytic conclusions. Adoption of the RRoC-STAR reporting standards is recommended for future comparative research.

Principle 4: Central lymph node metastasis in right colon cancer does not constitute incurable systemic disease. A clinical assumption that has historically discouraged aggressive surgical management of central nodal disease is that D3-positive right colon cancer represents systemic, and therefore incurable, disease. The prospective data from Banipal et al. [39] challenge this assumption: among 42 patients with histologically confirmed D3 metastases, those who underwent R0 resection (D3-RDN, n=29) achieved a five-year OS of 72.9% and DFS of 73.1%, substantially superior to the historically cited five-year OS of 35-48% [57], with survival improving across successive time periods, consistent with a learning-curve effect [41]. Long-term outcome data from D3 dissection in Stage II/III right-sided colon cancer support similar conclusions [58,59].

This is further contextualized by the lymph node mapping data of Kataoka et al. [10]: in right colon cancer, L3 (central) node involvement does not carry the same independent adverse prognostic weight as in left-sided disease, being associated with worse DFS but not OS, in contrast to the significant OS impairment for L3-positive left colon cancers. This side-specific difference suggests that central nodal spread in right colon cancer may more frequently represent locoregional rather than systemic dissemination, potentially amenable to surgical cure when R0 resection is achieved [60]. We emphasize, however, that this inference rests substantially on observational and subgroup data and remains susceptible to selection bias, stage migration, and learning-curve confounding; it is therefore biologically plausible and supported but not definitively established.

GRADE LOW: R0 resection of D3-positive right colon cancer achieves curative outcomes in a majority of patients in observational series. The learning-curve effect is documented, and centralization to experienced centers is supported.

Principle 5: Institutional experience and centralization are determinants of outcome. Extended lymphadenectomy for right-sided colon cancer is a technically demanding procedure with a substantial and well-documented learning curve. Available data suggest that laparoscopic proficiency requires approximately 20-30 or more supervised procedures even for experienced colorectal surgeons, with several series describing continued improvement beyond this range [27,41]. The survival improvement across successive time periods documented by Banipal et al. [39] is consistent with this learning curve and implies that outcomes in earlier series may underestimate the benefit achievable with mature expertise. Grüter et al. [47] demonstrated improved surgical quality and oncological outcomes following national implementation of a standardized CME technique, with centralization of complex cases serving as a key driver of improvement, although feasibility has also been demonstrated in lower-volume settings with appropriate training [61]. Robotic and minimally invasive approaches may further standardize the procedure [62-66]. An evidence-based minimum annual institutional volume has not, however, been formally established, and we identify this as a research priority.

These observations have direct practical implications. The increased vascular injury risk associated with extended lymphadenectomy, approximately 3% in experienced RELARC centers versus 1% in D2 arms [16], is not an intrinsic and immutable property of the technique but an experience-dependent complication that decreases as surgical volume increases [45,67,68]. Restricting extended lymphadenectomy to centers with documented proficiency is, therefore, an evidence-based quality standard analogous to the centralization of complex hepatobiliary and esophagogastric surgery [47,69].

GRADE MODERATE: Institutional experience is an independent determinant of outcome. Centralization to high-volume, experienced centers is supported by learning-curve and quality-improvement data.

Why Seven Meta-Analyses Could Not Resolve This Clinical Question

The existence of multiple published systematic reviews and meta-analyses reaching diametrically opposite conclusions on the same clinical question is itself an informative meta-epidemiological finding [25-31,54]. This review concludes that additional statistical pooling of this particular, heterogeneous observational evidence base, rather than meta-analysis as a method, amplifies rather than resolves clinical uncertainty, because the relevant sources of heterogeneity are not amenable to statistical correction. These include inconsistent surgical definitions, what constitutes “D3” varies from SMV-level dissection in Japanese series [5] to full bilateral SMA clearance in some Western centers [4]; pseudo-replication from overlapping patient cohorts reported across multiple publications from the same databases; secular trends in surgical experience and adjuvant chemotherapy confounding historical comparisons [43]; and biological heterogeneity within “right-sided colon cancer,” a designation that conflates cecal cancers fed by the ICA with hepatic flexure and transverse colon cancers in the MCA territory, which exhibit profoundly different D3 metastasis rates [10,11]. Meta-analysis remains a valuable method where these conditions are met; the limitation lies in this evidence base, not in the technique.

The Cochrane Handbook guidance [36] invoked in our Methods is directly applicable: when substantial clinical, methodological, and statistical heterogeneity renders pooled analysis misleading, structured narrative synthesis with explicit GRADE assessments [35] provides a more honest and actionable summary of the evidence than a numerical meta-analytic estimate that conveys spurious precision.

PROSPERO Registration and GRADE Assessments: Methodological Transparency

The PROSPERO registration (CRD420261334953) and pre-specified application of RoB-2 [32], ROBINS-I [33], and AMSTAR-2 [34] distinguish this review from the majority of prior systematic analyses in this field. Of seven identified meta-analyses, five were rated Low or Critically Low quality by AMSTAR-2, with common deficiencies including absence of a pre-registered protocol (5/7) and inadequate exploration of heterogeneity despite high I² values (37-96%) across studies. These shortcomings explain, at least partially, why prior reviews generated confusion rather than clinical guidance.

Our GRADE certainty profiles, ranging from VERY LOW for observational survival outcomes to MODERATE for complication rates, reflect the current state of evidence with appropriate epistemic transparency [35]. The highest certainty achieved, LOW, for the lymph node yield finding appropriately acknowledges that increased nodal harvest is consistently demonstrated, but its survival significance is unproven. Reporting GRADE certainty alongside every outcome is the minimum standard for systematic reviews informing clinical practice and a prerequisite for meaningful guideline development [69,70].

Evidence-Based Patient Selection Framework

Integrating the prospective RCT data, lymph node mapping studies [10,11], meta-analytic evidence [25-31], and surgical quality literature synthesized in this review, we propose a three-tier patient selection framework for extended lymphadenectomy in right-sided colon cancer (Table 4). This framework is an interpretative, evidence-informed synthesis intended to support individualized, multidisciplinary decision-making; it is not a guideline-level recommendation and requires prospective validation before widespread adoption. It is distinguished from prior proposals [27,71] by its explicit incorporation of feeding artery anatomy, GRADE certainty ratings for each recommendation, and alignment with preoperative CT-based nodal assessment as a decision trigger [8,72].

**Table 4:** Evidence-informed three-tier patient selection framework for extended lymphadenectomy in right-sided colon cancer (interpretative; requires prospective validation) ASA: American Society of Anesthesiologists; CME: complete mesocolic excision; CT: computed tomography; CVL: central vascular ligation; DFS: disease-free survival; MCA: middle colic artery; MDT: multidisciplinary team; OS: overall survival; RELARC: Radical Extent of Lymphadenectomy of LAparoscopic Right Colectomy The framework is interpretative and requires prospective validation; GRADE ratings indicate the certainty of the supporting evidence for each tier.

Tumor category	Recommended approach	Key evidence	GRADE
TIER 1-Standard D2 lymphadenectomy (routine CME/D3 not indicated)			
Cecum, all stages (any T, cN0 or cN+)	Standard D2 (≥12 nodes)	D3 metastasis 3.8% (T-REX). SMA dissection is not required.	MODERATE
Ascending colon, Stage I–II (T1–T2, cN0)	Standard D2 (≥12 nodes)	D3 metastasis 1.1% (T-REX). No DFS/OS benefit Stage I–II in RELARC.	MODERATE
TIER 2-Selective CME + D3 (high-risk features, experienced center)			
Ascending colon, Stage III (T3–T4, cN+ / pN2)	CME with CVL + D3 (experienced center)	RELARC subgroups (exploratory): pN2 HR 0.25, LVI+ HR 0.34. Banipal: D3-RDN R0 5-yr OS 72.9%.	LOW
Hepatic flexure & transverse colon, Stage II–III	CME with CVL + D3 (complete MCA dissection)	T-REX MCA-fed tumors: 6.7–7.5% D3 metastasis. Kataoka: L3 metastasis 8.5% in the right colon.	LOW
TIER 3 — Individualized / expert-centered decision			
Any location with preop CT central nodes >10 mm	Individualized CME+D3; MDT discussion	Banipal: preop CT detects D3 in ~50% of D3+ cases. Long: nomogram-guided selection.	MODERATE
Elderly (≥80 y) or ASA ≥3	Standard D2 (comorbidity-adjusted)	Increased morbidity without OS benefit in frail patients. Lygre: complications rise with age and comorbidity.	MODERATE

Preoperative Staging, Quality Assurance, and Future Priorities

Translation of this framework into routine practice requires two currently underdeveloped capabilities: accurate preoperative identification of patients at high risk for central nodal involvement and objective intraoperative and postoperative assessment of surgical quality.

Preoperative staging: Current CT imaging achieves only moderate sensitivity (~72%) for detecting central node involvement [39]. High-resolution CT angiography with 3D vascular reconstruction [8,73] and indocyanine green fluorescence-guided lymphatic mapping [74] represent promising tools for preoperative risk stratification, complementing earlier work on sentinel lymph node mapping in colorectal cancer [74]. Predictive nomograms based on clinicopathological features may aid case selection [71]. Prospective validation of CT-based nodal criteria as triggers for referral to experienced centers and extended dissection would represent a significant clinical advance.

Quality assurance: The Benz classification [18], photographic specimen documentation, and systematic measurement of vessel stump lengths [44] and mesenteric area [21] constitute the minimum quality metrics that should be recorded in all centers performing extended lymphadenectomies. Visual analog scales for reporting the extent of lymphadenectomy [51] may further standardize documentation. Prospective registries with mandatory quality reporting, modeled on the national implementation studied by Grüter et al. [47], would enable ongoing monitoring of outcomes and identification of learning-curve thresholds.

Future research priorities: These include mature primary endpoint data from COLD, CoME-in, and RICON; prospective validation of pN2 and LVI+ as enrichment criteria for D3 benefit in dedicated trials; integration of molecular and genomic tumor profiling, including mismatch-repair/microsatellite instability status, BRAF and RAS mutational status, and consensus molecular subtypes, given the recognized biological differences between right- and left-sided tumors as potential effect modifiers influencing patient selection; universal adoption of RRoC-STAR reporting standards; formal health-economic evaluation of centralization policies; and functional outcomes assessment, including bowel function and nutritional status after extended dissection.

Limitations

This review has several limitations. Despite comprehensive database searching, publication bias may have favored positive results in the observational literature; formal funnel plot assessment was precluded by outcome heterogeneity. The narrative synthesis approach, while validated by the documented failure of seven prior meta-analyses to resolve this question through statistical pooling of this evidence base [25-31], lacks the numerical precision of quantitative estimates. The primary survival endpoint of RELARC, while not statistically significant, showed a clinically meaningful trend (HR 0.74 for DFS) [17], and the trial may have been underpowered to detect a modest true benefit in an unselected population. Long-term primary endpoint data from three of six prospective studies (COLD, CoME-in, RICON) are unavailable, making definitive survival conclusions premature. Molecular profiling information was inconsistently reported, precluding systematic subgroup analysis by tumor biology. Finally, significant heterogeneity in surgical definitions, partially addressed by the RRoC-STAR consensus [15] but not yet universally adopted, limits direct comparison across included trials. These limitations reinforce that the current evidence supports informed, individualized clinical decision-making rather than definitive guideline recommendations and that the question of extended lymphadenectomy for right-sided colon cancer remains genuinely and appropriately open.

## Conclusions

Extended lymphadenectomy consistently improves lymph node yield but has not demonstrated a universal survival benefit in prospective trials. The available evidence supports a stratified rather than universal approach: standard D2 dissection remains appropriate for cecal and early-stage ascending colon cancer, whereas selective CME with CVL and D3 dissection may be considered for Stage III disease and for hepatic flexure or transverse colon tumors with high-risk features, provided these procedures are performed in experienced centers with structured quality monitoring and multidisciplinary oversight. This selective recommendation is based on low- to very low-certainty evidence and should be regarded as evidence-informed rather than guideline-level. Central lymph node metastasis in right-sided colon cancer does not appear to represent invariably incurable systemic disease, and R0 resection can achieve favorable long-term outcomes in experienced hands, although this conclusion is derived largely from observational data. The clinical priority should be the standardization of surgical quality through objective specimen assessment, prospective registries, and appropriate centralization, rather than the uniform extension of dissection to all patients. Mature survival data from ongoing trials are required before definitive guideline recommendations can be made.

## Figures and Tables

**Figure 6:**
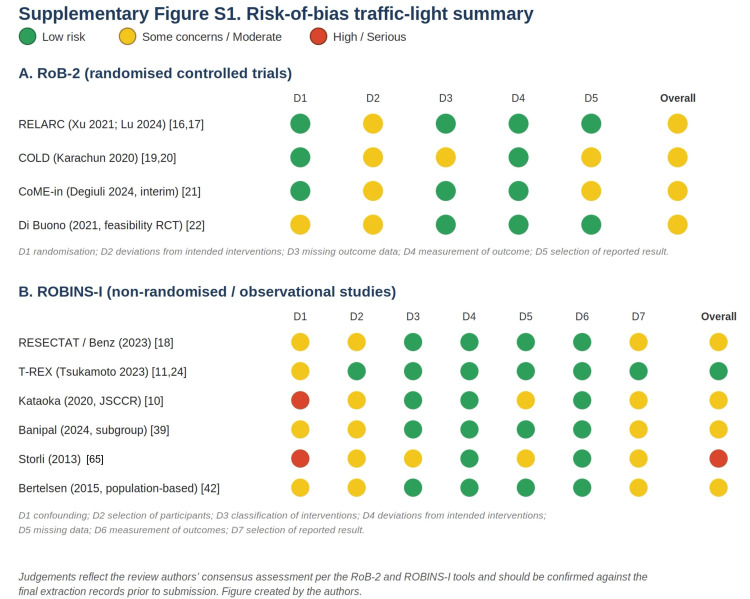
Risk-of-bias traffic-light summary Green=low risk; amber=some concerns (RoB-2)/moderate (ROBINS-I); red=high (RoB-2)/serious (ROBINS-I). [10,11,16-22,24,39,42,65]

## Appendices

Appendix 1

Full Electronic Search Strategies

Databases searched from inception through February 28, 2026, with no language restrictions. Strategies combine controlled vocabulary (MeSH/Emtree) and free-text terms across three concept blocks: right-sided colon cancer; extended lymphadenectomy / CME / CVL / D3; study design filters were not applied to maximise sensitivity. Records were de-duplicated in Covidence. [VERIFY against the strings exported from each database]

PubMed/MEDLINE

#1  "Colonic Neoplasms"[Mesh] OR (colon*[tiab] AND (cancer*[tiab] OR carcinoma*[tiab] OR neoplas*[tiab] OR adenocarcinoma*[tiab] OR tumour*[tiab] OR tumor*[tiab]))

#2  "right-sided"[tiab] OR right colon*[tiab] OR ascending colon*[tiab] OR caecal[tiab] OR cecal[tiab] OR caecum[tiab] OR cecum[tiab] OR hepatic flexure*[tiab] OR proximal colon*[tiab] OR transverse colon*[tiab]

#3  "Lymph Node Excision"[Mesh] OR lymphadenectomy[tiab] OR lymph node dissection*[tiab] OR ("complete mesocolic excision"[tiab] OR CME[tiab]) OR ("central vascular ligation"[tiab] OR CVL[tiab]) OR D3[tiab] OR "central lymph node*"[tiab] OR "extended lymphadenectomy"[tiab]

#4  #1 AND #2 AND #3

Result count exported to PRISMA Figure 2: PubMed/MEDLINE n=450 

Scopus

( TITLE-ABS-KEY ( colon*  W/3  ( cancer*  OR  carcinoma*  OR  neoplas*  OR  adenocarcinoma*  OR  tumour*  OR  tumor* ) ) )  AND  ( TITLE-ABS-KEY ( "right-sided"  OR  "right colon"  OR  "ascending colon"  OR  cecal  OR  caecal  OR  cecum  OR  caecum  OR  "hepatic flexure"  OR  "transverse colon" ) )  AND  ( TITLE-ABS-KEY ( lymphadenectomy  OR  "lymph node dissection"  OR  "complete mesocolic excision"  OR  CME  OR  "central vascular ligation"  OR  CVL  OR  D3  OR  "central lymph node"  OR  "extended lymphadenectomy" ) )

Scopus n=380 

Web of Science (Core Collection)

TS=(colon* NEAR/3 (cancer* OR carcinoma* OR neoplas* OR adenocarcinoma* OR tumo$r*)) AND TS=("right-sided" OR "right colon" OR "ascending colon" OR cecal OR caecal OR cecum OR caecum OR "hepatic flexure" OR "transverse colon") AND TS=(lymphadenectomy OR "lymph node dissection" OR "complete mesocolic excision" OR CME OR "central vascular ligation" OR CVL OR D3 OR "central lymph node" OR "extended lymphadenectomy")

Web of Science n=420.

S1.4 Cochrane Central Register of Controlled Trials (CENTRAL)

#1  MeSH descriptor: [Colonic Neoplasms] explode all trees

#2  (colon* NEAR/3 (cancer* OR carcinoma* OR neoplas* OR adenocarcinoma* OR tumour* OR tumor*)):ti,ab,kw

#3  (right-sided OR "right colon" OR "ascending colon" OR cecal OR caecal OR cecum OR caecum OR "hepatic flexure" OR "transverse colon"):ti,ab,kw

#4  (lymphadenectomy OR "lymph node dissection" OR "complete mesocolic excision" OR CME OR "central vascular ligation" OR CVL OR D3 OR "central lymph node" OR "extended lymphadenectomy"):ti,ab,kw

#5  (#1 OR #2) AND #3 AND #4

Cochrane CENTRAL n=85. Total before de-duplication n=1,335; after 285 duplicates removed, 1,050 unique records screened.

Risk of Bias: RoB-2 (RCTs)

Domain-level judgements per the Cochrane RoB-2 tool. L=Low; SC=Some concerns; H=High. Surgical RCTs cannot blind operators; D2 (deviations) is rated “Some concerns” where this could affect delivery of the intended intervention.

**Table 5:** Risk of Bias-RoB-2 (randomized controlled trials) D1 randomization; D2 deviations from intended interventions; D3 missing outcome data; D4 measurement of the outcome; D5 selection of the reported result.

Trial	D1	D2	D3	D4	D5	Overall	Main reason
RELARC [16,17]	L	SC	L	L	L	SC	Open-label surgery; objective survival endpoints
COLD [19,20]	L	SC	SC	L	SC	SC	Only 100/768 reported; primary endpoint unpublished
CoME-in [21]	L	SC	L	L	SC	SC	Interim analysis; survival data pending
Di Buono [22]	SC	SC	L	L	L	SC	Single-centre feasibility RCT; short-term endpoints

Appendix 2

Risk of Bias: ROBINS-I (Non-Randomized/Observational Studies)

Domain-level judgements per ROBINS-I. L=Low; M=Moderate; S=Serious; C=Critical. Confounding (D1) is the predominant limitation in non-randomized comparisons of surgical extent.

**Table 6:** Risk of Bias-ROBINS-I (non-randomized/observational studies) D1 confounding; D2 selection of participants; D3 classification of interventions; D4 deviations from intended interventions; D5 missing data; D6 measurement of outcomes; D7 selection of the reported result.

Study	D1	D2	D3	D4	D5	D6	D7	Overall	Main reason
RESECTAT/Benz [18]	M	M	L	L	L	L	M	Moderate	Non-randomized; case-mix differences
T-REX [11,24]	M	L	L	L	L	L	L	Moderate	Prospective protocolized cohort; residual confounding
Kataoka [10]	S	M	L	L	M	L	M	Serious	Retrospective registry; selection by stage
Banipal [39]	M	M	L	L	L	L	M	Moderate	Subgroup of ongoing trial; small D3+ n
Storli [65]	S	M	M	L	M	L	M	Serious	Historical comparison; era confounding
Bertelsen [42]	M	M	L	L	L	L	M	Moderate	Population-based; non-randomized allocation

Appendix 3

Critical Appraisal: AMSTAR-2 (Systematic Reviews/Meta-Analyses)

Condensed AMSTAR-2 appraisal of the seven systematic reviews/meta-analyses [25-31], reporting the seven critical domains (items 2, 4, 7, 9, 11, 13, 15) and the overall confidence rating. Y=Yes; PY=Partial Yes; N=No; n/a=not applicable (narrative review).

**Table 7:** Critical appraisal - AMSTAR-2 (systematic reviews/meta-analyses) Summary: 2 Moderate, 2 Low, 3 Critically Low. Critical weaknesses concentrated in the absence of a pre-registered protocol (item 2), no list of excluded studies (item 7), and no assessment of publication bias (item 15), consistent with the manuscript narrative.

Review	2 (protocol)	4 (search)	7 (excl. list)	9 (RoB)	11 (meta)	13 (RoB in synth.)	15 (pub. bias)	Overall
De Simoni 2021 [25]	Y	Y	N	Y	Y	PY	N	Moderate
Hayashi 2024 [26]	Y	Y	N	Y	Y	Y	PY	Moderate
Gupta 2024 [27]	N	PY	N	N	n/a	N	N	Critically Low
Pompeu 2025 [28]	N	Y	N	PY	Y	PY	N	Low
Anania 2021 [29]	N	Y	N	PY	Y	N	N	Low
El-fatah 2023 [30]	N	PY	N	N	Y	N	N	Critically Low
Seretis 2025 [31]	N	PY	N	N	n/a	N	N	Critically Low

Risk-of-Bias Traffic-Light Summary

Appendix 4

Excluded Full-Text Studies With Reasons

Of 195 full-text articles assessed, 140 were excluded. The distribution by reason is shown below

**Table 8:** Excluded full-text studies

Reason for exclusion	n	Representative examples/notes
Wrong intervention (no extended/central dissection; comparison not D2 vs D3/CME)	45	Sun et al., 2021, Colorectal Disease, “Chylous ascites after complete mesocolic excision for right-sided colon cancer with D3 lymphadenectomy”: focused on a specific postoperative complication after CME/D3 rather than an eligible comparative assessment of extended versus standard lymphadenectomy.
Wrong population (not right-sided; rectal/left colon; benign; metastatic-only)	32	Feng et al., 2016, Journal of Laparoendoscopic & Advanced Surgical Techniques, “Laparoscopic Complete Mesocolic Excision for Stage II/III Left-Sided Colon Cancers”: excluded because the study population involved left-sided colon cancer rather than right-sided colon cancer.
No appropriate comparator group	28	Enomoto et al., 2021, Surgical Endoscopy, “Laparoscopic middle colic artery-preserved right hemicolectomy with true D3 lymph node dissection for right-sided colon cancer”: excluded because it described a modified D3/CME technique without an appropriate D2 or conventional right hemicolectomy comparator group.
Insufficient extractable outcome data	22	Xie et al., 2017, Annals of Surgical Oncology, “An Optimal Approach for Laparoscopic D3 Lymphadenectomy Plus Complete Mesocolic Excision (D3+CME) for Right-Sided Colon Cancer”: excluded because the report was primarily technical/video-based and did not provide sufficient extractable comparative outcome data by study arm.
Duplicate publication / overlapping cohort	13	Lu et al., 2016, Trials, “The RELARC trial: study protocol for a randomized controlled trial”: excluded as a protocol/preliminary report overlapping with the later RELARC trial outcome publication, retained for evidence synthesis.
Total excluded	140	

Appendix 5

Full GRADE Evidence Profile

Outcome-level certainty assessment across the five GRADE domains. Symbols: ⊕⊕⊕⊕ High; ⊕⊕⊕○ Moderate; ⊕⊕○○ Low; ⊕○○○ Very low. “nSerious”=not serious. This profile expands Table 3 of the main text.

**Table 9:** Full GRADE evidence profile DFS: disease-free survival; MA: meta-analyses; OS: overall survival; RCT: randomized controlled trial Imprecision for safety outcomes reflects low absolute event counts; indirectness for lymph node yield reflects its status as a surrogate not validated against survival in these trials.

Outcome (studies)	Risk of bias	Inconsistency	Indirectness	Imprecision	Other	Certainty
Lymph node yield (1 RCT + 7 MA)	nSerious	Serious (I²=96%)	Serious (surrogate)	nSerious	—	⊕⊕○○ LOW
3-yr DFS – RELARC primary (1 RCT)	nSerious	nSerious	Serious (single country)	Serious (CI crosses 1)	—	⊕⊕○○ LOW
3-yr OS (1 RCT)	nSerious	nSerious	Serious	Serious (CI crosses 1)	—	⊕⊕○○ LOW
5-yr OS (observational + MA)	Serious (confounding)	Serious (I² 37–96%)	nSerious	nSerious	Pub. bias suspected	⊕○○○ VERY LOW
Vascular injury (1 RCT + obs.)	nSerious	nSerious	nSerious	Serious (low events)	—	⊕⊕⊕○MODERATE
Overall complications (4 trials)	nSerious	nSerious	nSerious	Serious (low events)	—	⊕⊕⊕○MODERATE
30-day mortality (4 trials)	nSerious	nSerious	nSerious	Serious (very rare)	—	⊕⊕⊕○MODERATE
Stage III subgroup benefit (1 RCT subgroup + 1 obs.)	Serious	Serious (inconsistent)	Serious (exploratory)	Serious	—	⊕○○○ VERY LOW
